# Intra-operative frozen section diagnosis of the suspicious solitary pulmonary nodule, how can we improve the strike-rate?

**DOI:** 10.1186/1749-8090-8-S1-O224

**Published:** 2013-09-11

**Authors:** R Govindraj, F Chowdhry, K Ang, D Waller

**Affiliations:** 1 University Hospitals of Leicester, Glenfield Hospital, Leicester, UK

## Background

To justify our policy towards suspicious solitary pulmonary nodules of intraoperative frozen section (IOFS) and not percutaneous biopsy, we evaluated its effectiveness and aimed to identify preoperative factors which may reduce unnecessary surgery for benign lesions.

## Methods

67 (27%) of last 250 consecutive patients (36M:31F, mean age:67yrs) discussed in MDT, operated for suspected cancer, underwent VATS nodule excision and IOFS. We reviewed perioperative data to determine effectiveness and performed multivariate analysis to identify factors predictive of malignant IOFS.

## Results

The positive predictivity of IOFS was 100%. Benign (including carcinoid) result was obtained in 25 patients (37%) comprising Organizing pneumonia-6, Granulomatous inflammation-6, Hamartomata-6, Carcinoid-5, Non-granulomatous inflammation-1, Solitary Fibrous Tumour-1. Malignant diagnoses included Adenocarcinoma-28, Squamous cell carcinomas-11, Large cell-2, Small cell lung cancer-1. Preoperative CTPET was undertaken in 44 patients (66%) Non-NSCLC NSCLC p value n 25 42 Age 62[21] 71[13] 0.003 Male% 44 59.3 0.218 Smoker% 60 71.4 0.335 Hx of malignancy12 14.3 0.791 Location, % 0.58 RUL 40 47.6 RML 16 4.8 RLL 28 23.8 LUL 16 19 LLL 0 2.4 >1lobe 0 2.4 Size,mm 15[10.0] 20.0[10.0] 0.013 Spiculated,% 36 90.5 0.001 SUV 2.9[2.0] 5.7[8.0] 0.012 In multivariate analysis, malignant IOFS result was associated with older patients (OR 0.94), with larger (OR 0.92) and more spiculated (OR 0.76) lesions on CT.

## Conclusion

A more effective protocol of VAT excision with intraoperative diagnosis with a reduced number of benign results could be achieved by routine use of CTPET and a more conservative, surveillance approach to more rounded nodules smaller than 2cm.

